# *Butyrivibrio hungatei* MB2003 Competes Effectively for Soluble Sugars Released by *Butyrivibrio proteoclasticus* B316^T^ during Growth on Xylan or Pectin

**DOI:** 10.1128/AEM.02056-18

**Published:** 2019-01-23

**Authors:** Nikola Palevich, William J. Kelly, Siva Ganesh, Jasna Rakonjac, Graeme T. Attwood

**Affiliations:** a AgResearch Limited, Grasslands Research Centre, Palmerston North, New Zealand; b Institute of Fundamental Sciences, Massey University, Palmerston North, New Zealand; University of Bayreuth

**Keywords:** *Butyrivibrio*, CAZy, rumen, bacteria, genome analysis, pectin, sugar ABC transport system, transcriptome, xylan

## Abstract

Rumen bacterial species belonging to the genus *Butyrivibrio* are important degraders of plant polysaccharides, particularly hemicelluloses (arabinoxylans) and pectin. Currently, four species are recognized; they have very similar substrate utilization profiles, but little is known about how these microorganisms are able to coexist in the rumen. To investigate this question, Butyrivibrio hungatei MB2003 and Butyrivibrio proteoclasticus B316^T^ were grown alone or in coculture on xylan or pectin, and their growth, release of sugars, fermentation end products, and transcriptomes were examined. In monocultures, B316^T^ was able to grow well on xylan and pectin, while MB2003 was unable to utilize either of these insoluble substrates to support significant growth. Cocultures of B316^T^ grown with MB2003 revealed that MB2003 showed growth almost equivalent to that of B316^T^ when either xylan or pectin was supplied as the substrate. The effect of coculture on the transcriptomes of B316^T^ and MB2003 was assessed; B316^T^ transcription was largely unaffected by the presence of MB2003, but MB2003 expressed a wide range of genes encoding proteins for carbohydrate degradation, central metabolism, oligosaccharide transport, and substrate assimilation, in order to compete with B316^T^ for the released sugars. These results suggest that B316^T^ has a role as an initiator of primary solubilization of xylan and pectin, while MB2003 competes effectively for the released soluble sugars to enable its growth and maintenance in the rumen.

**IMPORTANCE** Feeding a future global population of 9 billion people and climate change are the primary challenges facing agriculture today. Ruminant livestock are important food-producing animals, and maximizing their productivity requires an understanding of their digestive systems and the roles played by rumen microbes in plant polysaccharide degradation. *Butyrivibrio* species are a phylogenetically diverse group of bacteria and are commonly found in the rumen, where they are a substantial source of polysaccharide-degrading enzymes for the depolymerization of lignocellulosic material. Our findings suggest that closely related species of *Butyrivibrio* have developed unique strategies for the degradation of plant fiber and the subsequent assimilation of carbohydrates in order to coexist in the competitive rumen environment. The identification of genes expressed during these competitive interactions gives further insight into the enzymatic machinery used by these bacteria as they degrade the xylan and pectin components of plant fiber.

## Abstract (precis)

Feeding a future global population of 9 billion people and climate change are the primary challenges facing agriculture today. Ruminant livestock are important food-producing animals, and maximizing their productivity requires an understanding of their digestive systems and the roles played by rumen microbes in plant polysaccharide degradation. *Butyrivibrio* species are a phylogenetically diverse group of bacteria and are commonly found in the rumen, where they are a substantial source of polysaccharide-degrading enzymes for the depolymerization of lignocellulosic material. Our findings suggest that closely related species of *Butyrivibrio* have developed unique strategies for the degradation of plant fiber and the subsequent assimilation of carbohydrates in order to coexist in the competitive rumen environment. The identification of genes expressed during these competitive interactions gives further insight into the enzymatic machinery used by these bacteria as they degrade the xylan and pectin components of plant fiber.

## INTRODUCTION

The microbial community responsible for degradation of plant fiber in the rumen is diverse (1–3), but the core rumen bacterial microbiome is composed of seven groups, i.e., *Prevotella*, *Butyrivibrio*, and *Ruminococcus*, as well as unclassified *Lachnospiraceae*, *Ruminococcaceae*, *Bacteroidales*, and *Clostridiales* (2). Only a few species within these groups possess the enzymatic machinery required to initiate the primary degradation of insoluble plant polysaccharides (4–10). Cross-feeding interactions are known to occur between the polysaccharide-degrading species and sugar-fermenting microbes, allowing a wider diversity of microorganisms to exist in the rumen (11). Bacterial species belonging to the genus *Butyrivibrio* are metabolically versatile (2) and efficiently utilize the insoluble complex polysaccharides xylan and pectin (4, 12–14). At present, there are four recognized *Butyrivibrio* species (15), but an understanding of the interactions among these species during the process of fiber degradation in the rumen (16–18) is lacking.

Comparisons of the genome sequences of Butyrivibrio hungatei MB2003 (19, 20) and Butyrivibrio proteoclasticus B316^T^ (14), and particularly their carbohydrate-active enzyme (CAZyme) profiles, show that these *Butyrivibrio* species are functionally similar, and they predict important roles for both of these species in the breakdown of hemicellulose and pectin. B316^T^ contains 342 predicted CAZymes and shows strong growth on both oat spelt xylan and apple pectin (21). However, although MB2003 encodes 225 CAZymes associated with hemicellulose and pectin degradation, phenotypic analysis showed that it cannot grow on these insoluble substrates (19, 20). Therefore, it is hypothesized that MB2003 relies on other rumen organisms with more developed polysaccharide-degrading abilities to initiate the degradation of insoluble substrates and competes for the released oligosaccharides and sugars to enable its growth. To investigate this idea, MB2003 and B316^T^ were grown on oat spelt xylan or apple pectin, separately in monocultures to compare their individual substrate utilization abilities and together in cocultures to investigate their interactions. Growth of the strains was followed with strain-specific quantitative PCRs (qPCRs), and monosaccharides released from these polymers and their subsequent utilization were measured, along with fermentation end products and gene transcript abundances.

## RESULTS

### *B. hungatei* MB2003 competes with *B. proteoclasticus* B316^T^ for sugars released from xylan and pectin.

**FIG 1:**
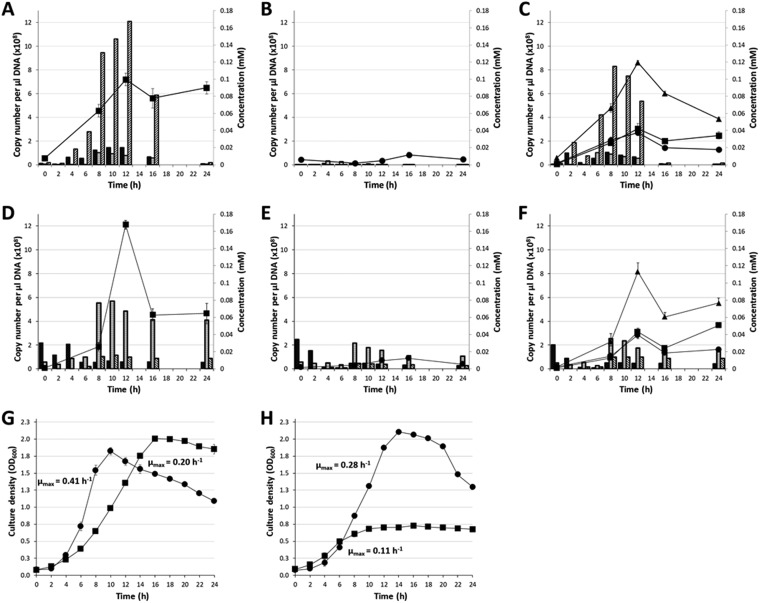
Growth and metabolite production of B. hungatei MB2003 and B. proteoclasticus B316^T^ grown on xylan and pectin. (A to C) Xylan-grown cultures and xylose (diagonally hatched bars), galactose (horizontally hatched bars), and arabinose (black filled bars) released by B316^T^ (black squares) in monoculture (A), MB2003 (black circles) in monoculture (B), and cocultures (black triangles) (C), as determined by qPCR. (D to F) Pectin-grown cultures and galactose (horizontally hatched bars), arabinose (black filled bars), and rhamnose (diagonally hatched bars) released by B316^T^ (black squares) in monoculture (D), MB2003 (black circles) in monoculture (E), and cocultures (black triangles) (F), as determined by qPCR. (G) Growth (OD_600_) of MB2003 (black circles) and B316^T^ (black squares) in medium containing component sugars of xylan from oat spelt as the substrates. (H) Growth (OD_600_) of MB2003 (black circles) and B316^T^ (black squares) in medium containing component sugars of apple pectin as the substrates. The maximum growth rate (μ_max_) was calculated and is reported per hour on each graph. Data are means of three replicates, and the error bars represent ±1 standard deviation of the mean.

Butyrivibrio hungatei MB2003 and Butyrivibrio proteoclasticus B316^T^ strains were grown as monocultures or cocultures on the insoluble substrates oat spelt xylan and apple pectin, to examine the interaction between the strains. The qPCR analysis of the monocultures showed that B316^T^ was able to utilize both xylan and pectin for growth (Fig. 1). MB2003 showed only slight growth on pectin and very little growth on xylan. In cocultures, MB2003 and B316^T^ displayed similar growth until 16 h when xylan or pectin was supplied as a substrate. B316^T^ grown in coculture with MB2003 on xylan or pectin showed greatly reduced growth, compared to its growth in monoculture.

Fermentation end products were used as an additional indicator of growth (see Table S1 in the supplemental material). Formate was the main volatile fatty acid (VFA) produced in B316^T^ monocultures and B316^T^ plus MB2003 cocultures on xylan, followed by butyrate and then acetate. In contrast, acetate was the main VFA produced by B316^T^ in monocultures and cocultures grown on pectin, followed by formate and butyrate. For MB2003 grown on xylan or pectin in monoculture, only small amounts of VFAs were produced, reflecting the poor growth of this strain alone on either of these insoluble substrates. The total amount of VFAs produced in pectin cocultures was less than the combined amounts of VFAs produced in MB2003 and B316^T^ monocultures, while cocultures grown on xylan produced more total VFAs than either of the monocultures.

Monosaccharides released from xylan and pectin during growth were measured using high-pressure ion chromatography (HPIC) (Fig. 1). For B316^T^ cells grown on xylan, the most abundant sugar detected was xylose. Maximum concentrations of xylose were detected at 12 h and 10 h in monoculture and coculture samples, respectively, with small amounts of arabinose and galactose being detected from 8 to 16 h of growth (Fig. 1A to C). The xylan-grown MB2003 monoculture samples showed very little release of monosaccharides (Fig. 1B). The pectin-grown MB2003 cultures showed similar sugar-release dynamics in the monoculture and coculture samples (Fig. 1E and F) but the monosaccharide detected at the highest concentration was galactose, with smaller amounts of arabinose and rhamnose. The B316^T^ monoculture produced a rapid release of galactose (0.08 mM) from 6 h to 8 h (Fig. 1D), while MB2003 released a smaller amount of galactose (0.03 mM) during the same phase of growth (Fig. 1E). A similar amount of galactose (0.04 mM) was released in the cocultures (Fig. 1F).

To investigate the relative abilities of MB2003 and B316^T^ to grow on the sugars released from xylan and pectin breakdown, these strains were grown as monocultures on mixtures of the component sugars of oat spelt xylan reflecting their abundance in the polymer (75 mol% xylose, 15 mol% glucose, and 10 mol% arabinose) and on similar mixtures for pectin (70 mol% galacturonic acid, 5 mol% glucose, 8 mol% galactose, 2 mol% arabinose, 9 mol% fucose, and 3 mol% rhamnose). The growth curves obtained for B316^T^ showed that it was readily able to utilize the sugar component mixtures of xylan and pectin for growth in monoculture, although the apple pectin component sugar mixture supported a lower maximum optical density at 600 nm (OD_600_) (Fig. 1G and H). MB2003 displayed faster rates of growth, compared to B316^T^, on both component sugar mixtures (0.41 h^−1^ versus 0.20 h^−1^ on xylan sugars and 0.28 h^−1^ versus 0.11 h^−1^ on pectin sugars) and also reached high culture OD_600_ values (Fig. 1G and H). Overall, these results indicate that B316^T^ is a primary degrader of both xylan and pectin, while MB2003 is able to compete effectively with B316^T^ for the released sugars by growing more quickly on the released sugars.

### Transcriptional changes and differential gene expression.

Samples of monocultures and cocultures of B316^T^ and MB2003 grown on xylan or pectin were collected at mid-log growth (12 h), and RNAs were extracted for transcriptome analyses (see Data Set S1 in the supplemental material). Transcripts were detected from a total of 1,896 genes in B316^T^ and 1,098 genes in MB2003 when grown on xylan and from 358 genes in B316^T^ and 3,000 genes in MB2003 when grown on pectin (Table 1). Among these transcripts, 1,300 genes in B316^T^ and 2,206 genes in MB2003 on both substrates had functional annotations. The total numbers of differentially expressed genes (DEGs) (false discovery rate [FDR] *Q* values of <0.05, Kruskal-Wallis test adjusted *P* values of <0.05, and ≥2-fold log_2_-transformed signal intensity differences) were 17 and 307 for the xylan growth condition and 56 and 787 for the pectin growth condition in B316^T^ and MB2003, respectively (Table 1). The most abundant DEGs (monoculture versus coculture) for B316^T^ transcripts were those associated with posttranslational modifications, carbohydrate metabolism (various CAZymes), sugar ABC transport systems, flagella biosynthesis, and some transposases (Data Set S1). In MB2003, the DEGs were associated with acetyl-coenzyme A (CoA) metabolism, sugar ABC transport systems, DNA replication, carbohydrate metabolism (various CAZymes), and flagella biosynthesis (Data Set S1). The full gene annotations, CAZy families, and clusters of orthologous groups (COG) functional assignments for all of the DEGs for B316^T^ and MB2003 cells grown in monocultures and cocultures on xylan and pectin are detailed in Data Sets S2 and S3.

**TABLE 1:** Differential gene expression analysis for B316^T^ and MB2003 grown on xylan and pectin

			No. of DEGs				
Sample and replicon	No. of ORFs in genome^*a*^	Total no. of ORFs mapped	FDR *Q* of <0.05^*b*^	Adjusted *P* of <0.05^*c*^	*Q*/*P* of <0.05 and log_2_ ≥2-fold changes	Monoculture^*d*^	Coculture^*d*^
Growth on xylan							
B316^T^							
Chromosome	2,939	1,582	152	66	14	8	6
pCY290	251	112	12	3	1	1	0
pCY360	425	151	10	4	1	0	1
pCY186	198	51	10	2	1	1	0
Total	3,813	1,896	184	75	17	10	7
MB2003							
Chromosome	2,767	878	501	413	279	189	90
BhuII	88	83	25	24	13	11	2
pNP144	147	137	30	24	15	15	0
pNP6	5	0	0	0	0	0	0
Total	3,007	1,098	556	461	307	215	92
Growth on pectin							
B316^T^							
Chromosome	2,939	257	167	42	42	38	4
pCY290	251	38	13	5	4	2	2
pCY360	425	45	45	9	9	8	1
pCY186	198	18	13	1	1	1	0
Total	3,813	358	238	57	56	49	7
MB2003							
Chromosome	2,767	2,765	986	671	659	604	55
BhuII	88	88	80	57	51	47	4
pNP144	147	147	129	79	77	74	3
pNP6	5	0	0	0	0	0	0
Total	3,007	3,000	1,195	807	787	725	62

a ORF, open reading frame.

b FDR reported as *Q* values.

c Kruskal-Wallis ANOVA, reported as *P* values.

d Numbers of DEGs were calculated by considering the monoculture and coculture conditions separately.

**FIG 2:**
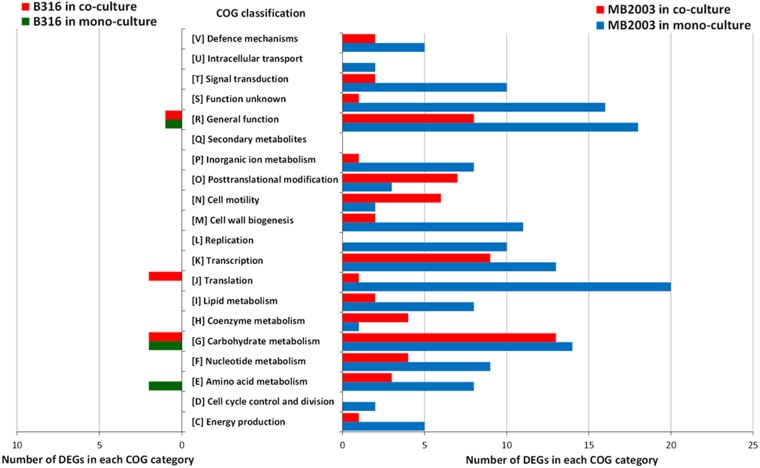
COG classifications of DEGs from B. proteoclasticus B316^T^ and B. hungatei MB2003 grown in monoculture and coculture on xylan. The analysis includes all DEGs with COG classifications.

**FIG 3:**
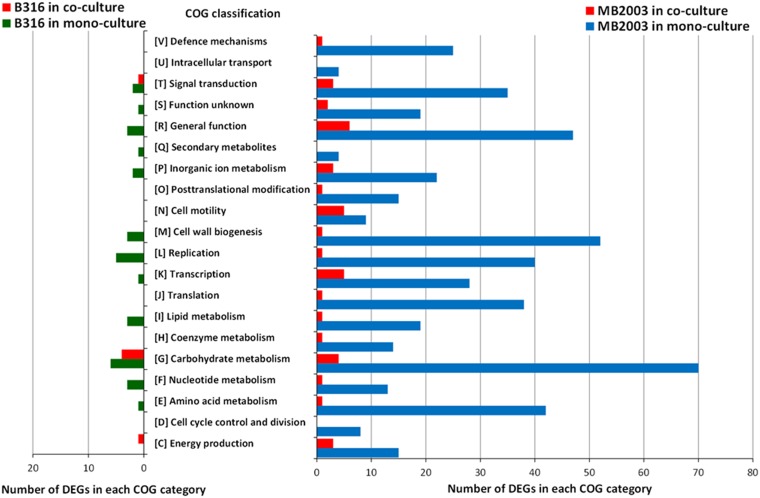
COG classifications of DEGs from B. proteoclasticus B316^T^ and B. hungatei MB2003 grown in monoculture and coculture on pectin. The analysis includes all DEGs with COG classifications.

Unusually, MB2003 grown on pectin had 107 genes upregulated in the monoculture that were not expressed at all in the coculture, while no DEGs were expressed exclusively in the coculture (Data Set S3). COG classifications were assigned to 56% and 66% of B316^T^ DEGs from cells grown on xylan and pectin, respectively, while 75% and 63% of MB2003 DEGs from cells grown on xylan and pectin, respectively, were assigned to COG categories. For MB2003 grown on xylan, the number of coculture DEGs assigned to the carbohydrate metabolism category was similar to the monoculture value, while the number of coculture DEGs assigned to the cell motility COG category was 3 times higher than the monoculture value (Fig. 2). This finding suggests that MB2003 upregulates its motility functions when cocultured with B316^T^ on xylan. For the pectin-grown cultures of MB2003, DEGs belonging to every COG category analyzed were upregulated to a significant extent in the monoculture, compared to the coculture (Fig. 3), indicating an effort to activate its metabolic capabilities in an attempt to support growth.

### Genes predicted to encode secreted CAZymes are differentially expressed during xylan and pectin degradation.

**FIG 4:**
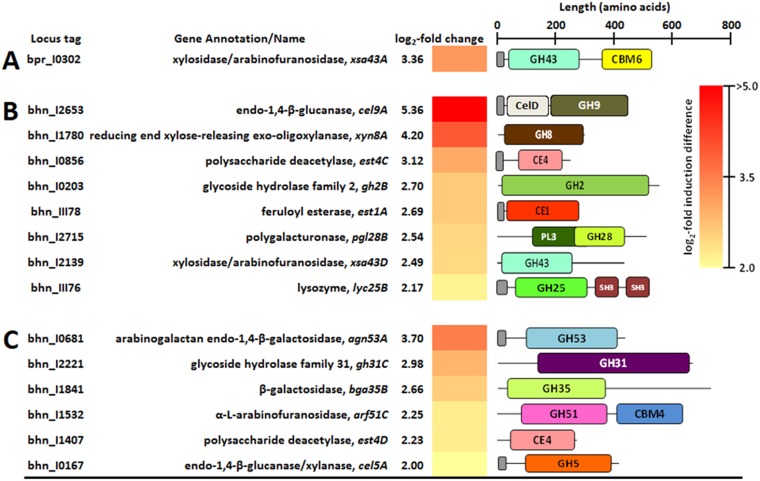
CAZyme-encoding DEGs upregulated in xylan-grown cultures. (A) B316^T^ grown in monoculture. (B) MB2003 grown in monoculture. (C) MB2003 grown in coculture. Small gray bars indicate signal peptide sequences.

Transcriptional analysis of DEGs encoding CAZymes indicated differences in carbohydrate metabolism of monocultures and cocultures of B316^T^ and MB2003 grown on xylan or pectin (Tables 2 and 3; also see Data Sets S2 and S3). MB2003 cells grown in monocultures and cocultures on xylan had 14 CAZymes significantly upregulated (Table 2), whereas cells grown on pectin had 42 CAZymes significantly upregulated (Table 2). Strikingly, for B316^T^, only *xsa43A* (xylosidase/arabinofuranosidase) from xylan-containing monocultures was highly upregulated, with a log_2_ 3.36-fold change (Fig. 4A), signifying that this is an important enzyme for xylan degradation. The presence of a signal peptide sequence on the CAZyme genes indicates that their encoded enzymes are secreted. Previous functional characterization of the *xsa43E* gene from B316^T^ found that this enzyme has dual β-xylosidase and α-l-arabinofuranosidase activities (22) and encodes an N-terminal glycoside hydrolase 43 (GH43) (Pfam04616) catalytic domain and a C-terminal carbohydrate-binding module 6 (CBM6) (Pfam03422) noncatalytic module that has been shown to bind xylan in other organisms (23). CBM6 domains are able to recognize xylose either as a monosaccharide, at the nonreducing end of xylo-oligosaccharides, or within the side chain components of xyloglucan (24). Several CBM6 modules also recognize (1,3)-β-d-linkages at the nonreducing end of β-glucans (25, 26) and appear to have coevolved with their associated catalytic domains to acquire the same substrate specificity (27). The α-l-arabinofuranosidases cleave arabinose side chains from substituted xylo-oligosaccharides derived from xylan (28). It is thought that the B316^T^ Xsa43A enzyme is secreted into the extracellular environment and has a role in disrupting the interpolymer and intrapolymer linkages within the xylan components of the plant cell wall.

**TABLE 2:** List of CAZymes significantly upregulated during monoculture and coculture growth of B. hungatei MB2003 on xylan^*a*^

Locus tag	Name	Annotation	CAZy	FDR	log_2_ fold change
MB2003 monoculture growth					
bhn_I2653	*cel9A*	Endo-1,4-β-glucanase	GH9/CelD	1.42E−77	5.36
bhn_I1780	*xyn8A*	Reducing-end xylose-releasing exo-oligoxylanase	GH8	7.38E−98	4.2
bhn_I0856	*est4C*	Polysaccharide deacetylase	CE4	1.57E−05	3.12
bhn_I0203	*gh2B*	Glycosyl hydrolase family 2	GH2	4.01E−16	2.7
bhn_III78	*est1A*	Feruloyl esterase	CE1	1.17E−17	2.69
bhn_I2715	*pgl28B*	Polygalacturonase	PL3, GH28	1.03E−02	2.54
bhn_I2139	*xsa43D*	Xylosidase/arabinofuranosidase	GH43	9.88E−03	2.49
bhn_III76	*lyc25B*	Lysozyme	GH25, 2× SH3	1.73E−18	2.17
MB2003 coculture growth					
bhn_I0681	*agn53A*	Arabinogalactan endo-1,4-β-galactosidase	GH53	1.78E+10	3.7
bhn_I2221	*gh31C*	Glycoside hydrolase family 31	GH31	1.73E+40	2.98
bhn_I1841	*bga35B*	β-Galactosidase	GH35	9.70E+11	2.66
bhn_I1532	*arf51C*	α-l-Arabinofuranosidase	GH51/CBM4	4.33E+22	2.25
bhn_I1407	*est4D*	Polysaccharide deacetylase	CE4	5.31E−03	2.23
bhn_I0167	*cel5A*	Endo-1,4-β-glucanase/xylanase	GH5	4.19E−03	2

a Genes were considered significantly expressed if all three thresholds were met, i.e., FDR calculated using Benjamini-Hochberg *Q* values of <0.05, Kruskal-Wallis test *P* values of <0.05, and log_2_ ≥2-fold difference in expression.

**TABLE 3:** List of CAZymes significantly upregulated during monoculture and coculture growth of B. hungatei MB2003 on pectin^*a*^

Locus tag	Name	Annotation	CAZy	FDR	log_2_ fold change
MB2003 monoculture growth					
bhn_III64	*bgl3A*	β-Glucosidase	GH3	5.66E+53	6.13
bhn_III76		Lysozyme	GH25	2.98E+19	6.04
bhn_I2226	*cbp94A*	Cellobiose phosphorylase	GT36	1.10E+86	5.83
bhn_I0187	*gh115A*	α-Glucuronidase	GH115	4.39E+53	4.94
bhn_I1972	*aga36C*	α-Galactosidase	GH27	4.38E+28	4.54
bhn_I0084	*gh27A*	α-Galactosidase	GH27	8.45E+34	4.5
bhn_I0681	*agn53A*	Arabinogalactan endo-1,4-β-galactosidase	GH53	1.71E+22	4.36
bhn_I0307	*est1B*	Feruloyl esterase	CE1	2.56E+23	4.25
bhn_I0092	*gh105B*	Unsaturated rhamnogalacturonyl hydrolase	GH88/GH105	1.26E+16	4.15
bhn_I1604	*gh31A*	Glycoside hydrolase family 31	GH31	5.45E+35	4.14
bhn_I2738	*amy13A*	α-Amylase	GH13	7.67E+18	4.11
bhn_I2139	*xsa43D*	Xylosidase/arabinofuranosidase	GH43	9.78E+18	4.09
bhn_I1308	*aga27A*	α-Galactosidase	GH27	1.12E+16	4.02
bhn_I0286	*gh31B*	α-Glucosidase	GH31	4.66E+14	3.91
bhn_I0192	*xyl3A*	β-Xylosidase	GH3	4.58E+12	3.85
bhn_I0183		Acetyl-xylan esterase	GH2/DUF303	9.96E+15	3.77
bhn_I2771	*gh30A*	Glycoside hydrolase family 30	GH30	5.91E+13	3.68
bhn_I0654	*aga36A*	α-Galactosidase	GH27	5.20E+10	3.56
bhn_I0680	*amy13D*	α-Amylase	GH13	1.39E+06	3.51
bhn_I2238	*pbp1A*	Penicillin-binding protein 1A family	GT51	8.06E+09	3.51
bhn_I0169	*xsa43A*	Xylosidase/arabinofuranosidase	GH43/CBM6	1.89E+09	3.41
bhn_I0840	*bga2C*	β-Galactosidase	GH2	4.62E+06	3.3
bhn_I1336	*glgX2*	Glycogen debranching enzyme	GH13	3.69E+06	3.27
bhn_I2065	*xsa43C*	Xylosidase/arabinofuranosidase	GH43	1.33E+06	3.26
bhn_I1873		Carbohydrate-binding protein	CBM2/6xCBM6	4.24E+09	3.24
bhn_I0185	*agu67A*	α-d-Glucuronidase	GH67	2.48E+08	3.23
bhn_I1407	*est4D*	Polysaccharide deacetylase	CE4	9.58E−05	3.21
bhn_I1967		Mannose-6-phosphate isomerase	GH1	2.31E+06	3.04
bhn_I2434	*est4B*	Polysaccharide deacetylase	CE4	8.83E−03	2.76
bhn_I0579	*xyn10B*	Endo-1,4-β-xylanase	GH10	6.33E−04	2.7
bhn_I2332	*pgl28B*	Polygalacturonase	GH28	1.31E−02	2.69
bhn_I1961	*cel5C*	Endo-1,4-β-glucanase	GH5/CBM2	1.35E−02	2.68
bhn_I1060	*amy13B*	α-Amylase	GH13	2.71E−02	2.56
bhn_I2290	*gh31C*	Glycoside hydrolase family 31	GH31	4.13E−02	2.44
bhn_I0644	*amy13B*	α-Amylase	GH13	3.49E−03	2.42
bhn_I2551	*bga2A*	β-Galactosidase	GH2	4.09E−04	2.41
bhn_I0194	*lyc25C*	Lysozyme	GH25	2.32E−02	2.13
bhn_I1969		Glycoside hydrolase GH130 family	GH130/GH43	2.15E−02	2.11
bhn_I2221	*gh31C*	Glycoside hydrolase family 31	GH31	6.89E−03	2.07
bhn_III72	*est4A*	Polysaccharide deacetylase	CE4	0.00E+00	N/A
MB2003 coculture growth					
bhn_I1532	*arf51C*	α-l-Arabinofuranosidase	GH51/CBM4	0.00E+00	5.05
bhn_I0847	*cel9B*	Cellodextrinase	GH9/CelD	1.74E−31	3.35

a Genes were considered significantly expressed if all three thresholds were met, i.e., FDR calculated using Benjamini-Hochberg *Q* values of <0.05, Kruskal-Wallis test *P* values of <0.05, and log_2_ ≥2-fold difference in expression.

Furthermore, high arabinofuranosidase activity was observed previously with Butyrivibrio fibrisolvens grown on xylan, suggesting that arabinofuranosidase activity may be regulated by the availability of arabinose (29–32). It was surprising that B316^T^ did not significantly upregulate any genes encoding secreted CAZymes when grown in monoculture or coculture on pectin; however, it displayed better growth in monoculture on pectin than on xylan (Fig. 1A and D).

**FIG 5:**
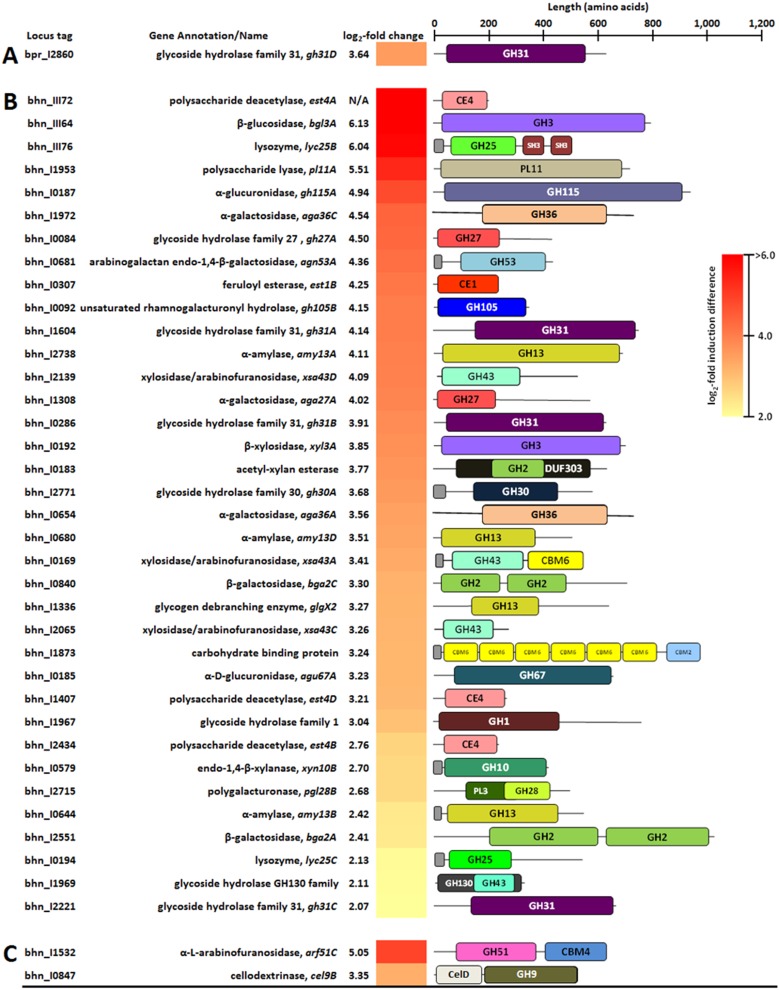
CAZyme-encoding DEGs upregulated in pectin-grown cultures. (A) B316^T^ grown in coculture. (B) MB2003 grown in monoculture. (C) MB2003 grown in coculture. signal peptide sequences. Small gray bars indicate signal peptide sequences.

In comparison, MB2003 expressed six CAZyme DEGs predicted to be present on the surface in the xylan-containing cultures and eight predicted to be present in the pectin-containing cultures (Fig. 4B and C and Fig. 5B and C). MB2003 xylan transcriptomes significantly expressed secreted enzymes containing carbohydrate esterase 1 (CE1), CE4, GH53, GH10, and GH5 CAZy domain-containing genes (Fig. 4B and C). The genes encoding extracellular feruloyl esterase (*est1A* [bhn_III78]) and polysaccharide deacetylase (*est4C* [bhn_I0856]), predicted to target the acetyl side groups of xylan, were significantly upregulated in MB2003 xylan monocultures (Fig. 4B; also see Data Set S1). Furthermore, in MB2003 xylan monocultures, *cel9A* (endo-1,4-β-glucanase [GH9/Pfam00759]) was the most upregulated CAZyme gene, with a log_2_ 5.36-fold change (Fig. 4B). In addition to the secreted enzymes containing the various GH and CE CAZy domains for degradation of glucuronoarabinoxylan (GAX) and its constituent side groups, the GH13 and pectate lyase 1 (PL1) domains target (1,4)-β-d-linkages within the galacturonic acid backbones, which are also necessary for the extracellular degradation of xylogalacturonan (XGA) and rhamnogalacturonan I (RG-I).

The eight genes encoding secreted CAZymes involved in pectin breakdown that were upregulated in MB2003 monoculture transcriptomes varied greatly in their predicted enzymatic capability (Fig. 5B). MB2003 significantly expressed secreted enzymes containing GH53, GH30, and GH13, as well as genes encoding GH43 and GH10 CAZy domains (Fig. 5B). Among these were genes with additional noncatalytic domains, including *lyc25B* encoding lysozyme (GH25 and two Src homology 3 [SH3] domains) and *xsa43A* encoding xylosidase/arabinofuranosidase (GH43 and CBM6 domains). The *xsa43A* gene of MB2003 is homologous to the *xsa43A* gene of B316^T^ (Fig. 4A) and is proposed to possess dual β-xylosidase and α-l-arabinofuranosidase activities. It is hypothesized that *xsa43A* is secreted in situations in which extracellular debranching of pectin results in release of arabinose and xylose side groups prior to pectic oligosaccharide assimilation.

The *gh30A* gene (bhn_I2771) was significantly upregulated only in MB2003 monocultures grown on pectin (Fig. 5B). This enzyme has a GH30 (Pfam02055) catalytic domain that is predicted to have activities in debranching of pectin to release d-xylose and to hydrolyze (1,4)-β-d-linkages in xylans (33), but their contribution to xylan and pectin degradation requires further biochemical verification. The β-1,4-galactanases containing GH53 catalytic domains are predicted to degrade galactan and arabinogalactan side chains in the hairy regions of pectin, by attacking the 1,4-β-d-galactosidic linkages in type I arabinogalactans (34). In MB2003, *agn53A* (bhn_I0681) was significantly expressed in pectin-grown monoculture cells and was the most upregulated gene in xylan-grown coculture cells (Fig. 4C and Fig. 5B). This finding suggests that *agn53A* encodes an important xylan- and pectin-degrading enzyme in MB2003. MB2003 contains one secreted carbohydrate-binding protein (CBP) (bhn_I1873; 984 amino acids), and this protein was significantly upregulated only in pectin-grown monoculture cells (Fig. 5B). The domain structure of bhn_I1873 is unique, containing six CBM6 domains toward the N terminus and a single C-terminal CBM2a domain. We propose that the significantly upregulated genes encoding the aforementioned enzymes contribute to the surface trimming and depolymerization of complex xylans (predominantly GAX) or pectin (predominantly XGA and RG-I).

### Carbohydrate uptake systems are upregulated in response to growth on xylan and pectin.

**FIG 6:**
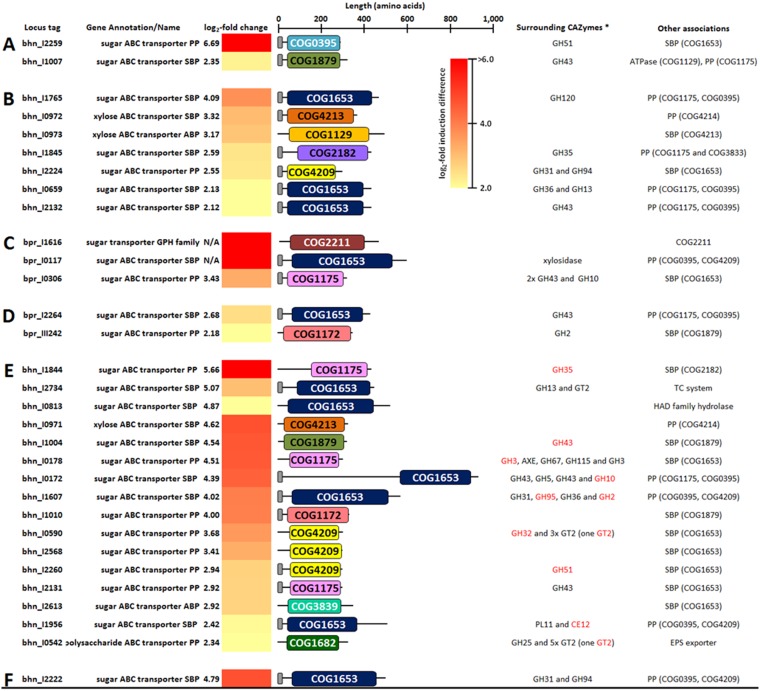
Functional domains of DEGs encoding carbohydrate transport proteins and surrounding CAZymes identified in xylan- or pectin-grown cultures. (A) MB2003 monoculture on xylan. (B) MB2003 coculture on xylan. (C) B316^T^ monoculture on pectin. (D) B316^T^ coculture on pectin. (E) MB2003 monoculture on pectin. (F) MB2003 coculture on pectin. *, CAZyme families of genes and DEGs (red) encoding polysaccharide-degrading enzymes colocalized with carbohydrate transport genes. SBP, solute-binding protein; PP, permease protein; AXE, acetyl-xylan esterase; ABP, ATP-binding protein; EPS, exopolysaccharide; TC system, two-component system histidine kinase; HAD family hydrolase, haloacid dehalogenase superfamily hydrolase. COG designations were as follows: COG0395, COG1175, COG1653, and COG1879, periplasmic component of ABC-type sugar transport system; COG1682, ABC-type polysaccharide/polyol phosphate export systems; COG2182, maltose-binding periplasmic proteins/domains; COG3833, permease component of ABC-type maltose transport systems; COG2211, Na^+^/melibiose symporter and related transporters; COG4213, periplasmic component of ABC-type xylose transport system; COG1129 and COG3839, ATPase component of ABC-type sugar transport systems; COG4209, permease component of ABC-type polysaccharide transport system; COG1172, permease component of ribose/xylose/arabinose/galactoside ABC-type transport systems. Small gray bars indicate signal peptide sequences.

Functional annotations of DEGs assigned to the COG category of carbohydrate transport and metabolism were investigated in order to determine which carbohydrates are taken up by B316^T^ and MB2003 cultures grown on xylan and pectin. The transcriptional analysis revealed a substantial number of ABC transporters upregulated in MB2003 grown on xylan or pectin in both cocultures and monocultures (Fig. 6). The most upregulated genes in both the xylan and pectin transcriptomes encode solute-binding proteins (SBPs) and permease proteins (PPs) of the sugar ABC transport systems. These proteins contained a carbohydrate uptake transporter type 1 (CUT1) domain (represented by COG1653 [SBPs] and COG0395, COG1175, and COG4209 [PPs]) or a CUT2 domain (COG1879 and COG4213 [SBPs] and COG1175 and COG4214 [PPs]). The CUT1 family mediates disaccharide and oligosaccharide uptake (35, 36).

Overall, the transcriptomic analysis identified upregulation of numerous genes encoding membrane proteins predicted to function as carbohydrate transporters (Data Sets S1 and S2). Among significantly upregulated genes encoding transporter proteins in MB2003, several genes have specific functions as xylose ABC transporters. For B316^T^, the only DEGs encoding carbohydrate transporters were predicted to function as general sugar ABC transporters.

DEGs associated with SBPs and PPs were identified in the pectin-grown B316^T^ transcriptomes only, with predicted functions as sugar ABC transporters (three in monoculture and two in coculture) (Fig. 6C and D). Signal peptides were predicted for the majority of the upregulated sugar ABC transporter SBPs and PPs, as well as the xylose transporter SBPs, in MB2003. Overall, these results emphasize the importance of SBP-dependent ATP-driven active transport of oligosaccharides and monosaccharides for growth in B316^T^ and MB2003.

### Coexpression of CAZyme genes associated with highly expressed genes encoding ABC transporter binding proteins.

The polysaccharide-degrading enzymes found adjacent to the SBPs include a number of xylosidases/arabinofuranosidases (GH43), endo-1,4-β-xylanase (GH10), and a β-mannosidase (GH2). MB2003 cells grown on xylan upregulated nine genes associated with sugar ABC transport systems, while 17 genes were upregulated on pectin (Fig. 6). Examples of the surrounding polysaccharide-degrading enzymes found in MB2003 xylan**-**grown cells include α-l-arabinofuranosidase (GH51), xylosidases/arabinofuranosidases (GH120 and GH43), α- and β-galactosidases (GH35 and GH36), sucrose phosphorylase (GH13), and glycosidases (GH31) (Fig. 6A and B). MB2003 grown in coculture on xylan upregulated bhn_I0972 and bhn_I0973, associated with xylose transport, while bhn_I0971 was upregulated in monoculture on pectin (Fig. 6B and E). All of the SBPs represented by the COG1653 domain in both B316^T^ and MB2003 were found proximal to other genes predicted to be involved in xylan degradation and metabolism, including various genes encoding GH43, GH10, GH51, GH53, and CE12 CAZy domains (Fig. 6). These included two SBP-encoding genes identified in xylan-grown B316^T^ monocultures and cocultures, bpr_I0182 and bpr_I0313, which were found previously in B316^T^ xylan-grown cells as part of polysaccharide utilization loci (14) predicted to be involved in hemicellulose degradation. The polysaccharide utilization loci containing these SBPs have genes encoding β-xylosidases, α-glucuronidases, acetyl-xylan esterases, ferulic acid esterases, and secreted endo-1,4-β-xylanases (33).

Similarly, several polysaccharide-degrading enzymes were identified proximal to the genes upregulated in MB2003 pectin transcriptomes. Examples include xylosidases/arabinofuranosidases (GH43 and GH51), endo-1,4-β-xylanase (GH10), acetyl-xylan esterase, endo-1,4-β-glucanase/xylanase (GH5), β-glucosidase (GH31), α-d-glucuronidase (GH67), α-glucuronidase (GH115), β-xylosidase (GH30), pectin esterase (CE12), and rhamnogalacturonan lyases (PL11) (Fig. 6E). The CAZymes surrounding the various SBPs were highly upregulated, and many of these were expressed only in the monoculture MB2003 transcriptomes (Data Set S3). In contrast, only one gene (bhn_I2222) was significantly upregulated in coculture, in proximity to a glucosidase (GH31) and cellobiose phosphorylase (GH94) (Fig. 6F). These findings are consistent with the extracellular breakdown of xylan and pectin by B316^T^, followed by capture and uptake of monosaccharides, xylo-oligosaccharides, and pectic oligosaccharides by the ABC transporters of both B316^T^ and MB2003.

### Debranching of xylo-oligosaccharides and pectic oligosaccharides is predicted to occur inside the cell.

The polysaccharide-degrading enzymes of MB2003 and B316^T^ both appear to be configured to carry out limited extracellular degradation of xylan and pectin. Xylo-oligosaccharides and pectic oligosaccharides entering the cytoplasm are predicted to be degraded by a range of exo-acting enzymes. Surprisingly, B316^T^ significantly upregulated only *gh31D* (a possible α-glucosidase [EC 3.2.1.20] or α-xylosidase [EC 3.2.1.-]) when grown in cocultures on pectin (Fig. 5A). In xylan-grown MB2003 transcriptomes, the two most upregulated cytosolic CAZyme genes were *xyn8A* (reducing-end xylose-releasing exo-oligoxylanase [GH8/Pfam01270]) and *est4C* (polysaccharide deacetylase [CE4/Pfam01522]) (Fig. 4B). The CAZyme genes upregulated in MB2003 monocultures grown on pectin varied greatly in their predicted enzymatic capabilities (Fig. 5B). These included genes with multiple domains (acetyl-xylan esterase containing a GH2 sugar-binding domain and a domain of unknown function [DUF303] [Pfam03629]), genes containing two identical catalytic domains within the same gene (β-glucosidase *bgl3D* with two GH3 domains [Pfam00933]), and two β-galactosidase genes (*bga2A* and *bga2C*) that each contain two GH2 (Pfam00703) domains. A polysaccharide lyase gene, *pl11A* (PL11/COG14111 domains), was also highly upregulated in the MB2003 pectin-grown monocultures, as were *lyc25B,* a lysozyme containing GH25 and two SH3 domains, and *xsa43A,* a xylosidase/arabinofuranosidase containing GH43 and CBM6 domains.

## DISCUSSION

Xylan and pectin are abundant plant structural polysaccharides and are major sources of energy for microbial fermentation within ruminants. The xylan derived from oat spelt that was used in this experiment is largely composed of GAX with a xylose backbone (37), while the apple pectin is mainly RG-I and XGA (38), with backbones of galacturonic acid and rhamnose (39). Rumen bacteria degrade xylans to xylose and arabinose, whereas pectins are degraded predominantly to rhamnose and galactose, along with a variety of oligosaccharides produced from both substrates (40–42). In the present study, B. proteoclasticus B316^T^ was confirmed to be a strong degrader of xylan and pectin, while B. hungatei MB2003 was unable to utilize either substrate alone to support significant growth (Fig. 1). MB2003 was able to grow only in cocultures with B316^T^, utilizing the sugars released by B316^T^ from xylan or pectin. The relationship between MB2003 and B316^T^ is not simple commensalism, as the growth of B316^T^ cells in cocultures with MB2003 was compromised, compared to their growth in monoculture. This is borne out by the growth of each strain on the sugar components of both xylan and pectin (Fig. 1G and H), which clearly demonstrated that MB2003 was able to achieve a higher growth rate than B316^T^ in both situations. This relationship appears to be a form of competition in which B316^T^ acts as a primary degrader of the insoluble substrates, releasing soluble sugars for its growth. MB2003 is able to compete for the soluble sugars in a manner that allows it to grow to almost the same extent as B316^T^ in cocultures, presumably enabling its coexistence with B316^T^ in the rumen.

Analyses of the soluble sugars released during growth on xylan showed that fermentation in the B316^T^ monoculture and coculture samples was complete by 12 h, after which the levels of xylose decreased dramatically (Fig. 1A and C), compared to MB2003, which released very little soluble sugar from xylan (Fig. 1B). The total pool of xylose detected in the B316^T^ plus MB2003 cocultures on xylan was lower than that in the B316^T^ monoculture, and the xylose concentration at 12 h postinoculation was reduced (Fig. 1A). It is possible that the other sugars analyzed (or not detected) could have been released and used immediately (or at time points not analyzed), such that their concentrations in the samples did not appear to increase. However, this seems unlikely, given that xylan extracted from oat spelt is typically >70% xylose and <10% arabinose and <15% glucose (CAS no. 9014-63-5, product no. X0627; Sigma-Aldrich). The growth experiments with monocultures of B316^T^ and MB2003 using the sugar components of xylan corroborate the hypothesis of MB2003 cross-feeding on the released xylose (Fig. 1G).

In pectin-grown monocultures and cocultures, galactose was the main monosaccharide detected. B316^T^ grown in monoculture contained the highest galactose concentrations (Fig. 1D), which is consistent with B316^T^ being more efficient at breaking down pectin. MB2003 had very poor, if any, ability to utilize pectin (20) but in cocultures MB2003 could compete with B316^T^ for the released galactose. The growth experiments on the sugar components of pectin suggest it is likely that, in addition to galactose, galacturonic acid (not detected in the HPIC analysis) was released during hydrolysis of pectin and was used by MB2003 (Fig. 1H). In a previous study, B316^T^ grown on pectin (43) showed increases in rhamnose, arabinose, and other monosaccharides and disaccharides but not galacturonic acid or galactose. This was interpreted as indicating that B316^T^ had utilized the galacturonic acid and galactose rapidly, such that they did not accumulate in the cultures.

Taken together, the growth experiments showed that MB2003 was unable to utilize xylan or pectin when grown in monoculture but was capable of significant growth on the breakdown products of both substrates when cocultured with B316^T^. MB2003 appears to sustain itself in coculture until primary degradation of xylan and pectin by B316^T^ is under way, and then it competes for the uptake of released soluble carbohydrates. The B316^T^ transcriptome analysis showed that only genes required for substrate utilization were significantly upregulated (Fig. 2 and 3). Genome sequence information (14) and proteome analyses (44, 45) indicate that B316^T^ primarily attacks the xylan backbone and substituent groups of hemicellulose outside the cell via secreted enzymes. The variable-length substituted or unsubstituted xylo-oligosaccharides are thought to be transported into the cell, where the final degradation occurs. B316^T^ most likely applies a similar approach for the degradation of pectin. The limited external breakdown of xylan or pectin, followed by the transport and utilization of xylo-oligosaccharides and pectic oligosaccharides in the cytoplasm, limits the loss of fermentable carbohydrates to competing bacterial species.

B. hungatei MB2003 cannot initiate the breakdown of either xylan or pectin but acts as a competitor for sugars released from the insoluble substrates through the primary degradation activity of B316^T^. MB2003 monocultures in xylan- or pectin-containing media strongly upregulated genes involved in almost every biological process, in an attempt to initiate growth. MB2003 grown in coculture with B316^T^ on xylan or pectin also showed upregulation of many genes, but to a lesser extent than in the monocultures. These upregulated genes include those encoding the enzymatic machinery required to utilize the oligosaccharides released from the initial degradation of xylan and pectin by B316^T^.

The B316^T^ and MB2003 gene complements suggest that they transport a variety of substituted xylan and pectin oligomers across their bacterial cell walls. *Butyrivibrio* species have Gram-positive cell wall structures (21), and monosaccharide transport across the bacterial cell wall is mediated by a variety of extracellular SBPs linked to dedicated sugar ABC transporter systems (19, 20). Recent data on the B316^T^ secretome (44, 46) and carbohydrate-transport-associated membrane proteins (45), as well as genome sequence analysis of MB2003 (19, 20), identified a large number of sugar-specific ABC transporter SBPs. Overall, analysis of MB2003 upregulated SBPs and their functional domains, along with surrounding genes encoding polysaccharide-degrading enzymes, revealed that the most prevalent functional category was the COG1653 domain, which is known to be associated with oligosaccharide transport (45). In B316^T^ and MB2003 pectin transcriptomes, there was upregulation of the genes predicted to encode sugar ABC transporter PPs (COG1172) and SBPs (COG1879), respectively. The encoded proteins have functional roles as ribose, xylose, arabinose, and galactose ABC transporters, suggesting a preference for these substrates. The substantial upregulation by MB2003 of sugar ABC transport system genes and a large variety of colocalized genes encoding polysaccharide-degrading enzymes (Fig. 6) supports the findings that MB2003 is able to grow only in coculture on xylan and pectin through cross-feeding on the released oligosaccharides and monosaccharides, such as xylose, arabinose, and rhamnose.

Once transported inside the cell, the oligosaccharides are degraded to their constituent monomers through the activity of several classes of cytosolic enzymes, including β-xylosidases, α-galactosidases, and α-glucuronidases that contain GH3, GH27, GH115, and GH67 CAZy domains (47–50). MB2003 in monocultures on pectin significantly expressed intracellular genes encoding enzymes containing GH3, GH27, GH115, and GH67 CAZy domains (Fig. 5B). In addition, MB2003 genes containing GH13 and PL11 CAZy domains (Fig. 6E) were expressed only in pectin-grown cells. This implies that the availability of a particular carbon source causes activation of a wider network of genes that enable these rumen bacteria to break down, to transport, and to metabolize such substrates for growth.

It is interesting to note that gene expression in B316^T^ was mostly unaffected by the presence of MB2003. However, the transcription levels of many genes encoding CAZymes, including the endo-1,4-β-xylanase genes *xyn10D* and *xyn10E* (bpr_I1083 and bpr_I1740) and the endo-1,4-β-glucanase gene *cel5D* (bpr_I0728), as well as sugar ABC transport system genes, were elevated in B316^T^ (Data Set S1).

It was observed that a MB2003 gene encoding a CBP was significantly upregulated only in pectin-grown monoculture cells. CBPs containing multiple noncatalytic domains have a possible role in a process called amorphogenesis (51), in which the interface between plant polysaccharides is interrupted by the binding of these proteins. Also, recent studies have shown that, in discrete regions of plant cell walls, initial enzymatic attack of pectin increases the access of CBMs to cellulose (52), effectively loosening the polysaccharide interactions to reveal the xylan and xyloglucan substrates (53, 54). In the rumen, B316^T^ and MB2003 may secrete these noncatalytic CBPs, along with polysaccharide-active enzymes, as a mechanism to enhance the rate and extent of plant cell wall degradation by disrupting the interface between other polysaccharides. The CBMs appear to be an important feature of noncatalytic CBPs from rumen *Butyrivibrio* (55) and therefore are of interest for future studies.

The transcriptome analyses have highlighted the differences in gene expression between B. proteoclasticus B316^T^ and B. hungatei MB2003 grown in monocultures and in cocultures, and they support the observed competitive interactions between these two rumen bacteria. B316^T^ showed relatively little change in gene expression when cocultured with MB2003 on either xylan or pectin. In contrast, MB2003 showed poor utilization of either xylan or pectin for growth. These analyses also highlight the diverse repertoire of *Butyrivibrio* genes encoding polysaccharide-degrading enzymes and sugar ABC transport systems that are expressed during xylan and pectin degradation. Noncatalytic proteins (e.g., CBPs) may also be important in this process. Future work should expand the biochemical characterization of enzymatic activities of the numerous CAZymes encoded in a wider range of *Butyrivibrio* species and should define the substrate specificities of their various SBPs for xylo-oligosaccharide and pectic oligosaccharide transport systems. This information will allow a better appreciation of the potential interactions that occur between *Butyrivibrio* species and an improved understanding of the degradation of insoluble substrates in the rumen.

## MATERIALS AND METHODS

### DNA sequencing, genome analysis, and curation.

B. hungatei MB2003 and B. proteoclasticus B316^T^ (56) were isolated from the rumen contents of fistulated Friesian dairy cattle (57) and sequenced (19, 20). DNA isolation and genome sequencing and assembly methods were described previously (20, 53). The GenBank accession numbers of the previously sequenced B. proteoclasticus B316^T^ nucleotide sequences (14) are CP001810 (main chromosome), CP001811 (BPc2), CP001812 (pCY360), and CP001813 (pCY186) (BioProject PRJNA29153).

### Growth of *Butyrivibrio* strains and isolation of RNA.

B. hungatei MB2003 and B. proteoclasticus B316^T^ were cultured anaerobically, under a CO_2_ atmosphere, at 39°C in prewarmed RM02 medium supplemented with 0.5% (wt/vol) cellobiose. When possible, the number of serial subculturing was reduced to a minimum before refreezing of stocks (−85°C), to avoid *in vitro* culture-biased evolution (58). Culture purity was verified via wet mounting and Gram staining. Cells were subcultured twice consecutively in triplicate with the respective growth medium, to ensure complete adaptation to the carbohydrate growth source. The OD_600_ was monitored using an Ultrospec 1100 Pro spectrophotometer (GE Healthcare, UK) over a period of 24 h. At mid-log phase of growth (OD_600_ of 0.4), cell counts for each monoculture were quantified using a Petroff-Hausser chamber (product no. 3900; Hausser Scientific, Horsham, PA, USA), according to the manufacturer’s instructions. Cell-density-equilibrated cultures were subcultured into 70 ml prewarmed RM02 medium with 0.5% (vol/vol) inoculum, supplemented with either 0.5% (wt/vol) xylan from oat spelt (CAS no. 9014-63-5, product no. X0627; Sigma-Aldrich, St. Louis, MO, USA) or pectin isolated from apple (CAS no. 9000-69-5, product no. 93854; Sigma-Aldrich) as the main carbohydrate source. For the monocultures, B316^T^ or MB2003 cells were inoculated separately into the medium; for the coculture samples, 0.25% (vol/vol) inocula of each equilibrated culture were added. Inoculations were done in triplicate for each organism (or coculture) and substrate, to be harvested at five time points (0, 8, 12, 16, and 24 h). On collection of each sample, 2 ml of each culture was removed and stored at −85°C for subsequent qPCR, HPIC, and VFA analyses. An overview of the MB2003 and B316^T^ coculture growth experiment is presented in Fig. S1 in the supplemental material. The remainder of each culture was snap frozen with liquid N_2_, and genomic RNA/DNA was extracted using a modified version of a liquid N_2_ and grinding method (59).

Briefly, 10 g of each frozen culture sample was combined with 2 volumes of RNA Bacterial Protect Reagent (Qiagen, Hilden, Germany). The thawed sample was centrifuged at 15,000 × *g* for 10 min at 4°C, and the cell pellet was resuspended in 1 ml of extraction buffer A (200 mM NaCl, 20 mM EDTA), 420 μl of 20% (wt/vol) SDS, and 1 ml of a mixture of phenol, chloroform, and isoamyl alcohol (25:24:1 [vol/vol/vol] [pH 4.5]; Thermo Fisher Scientific Inc., Waltham, MA, USA). The cells were disrupted by five rounds of bead beating, using a Mini-Beadbeater-96 (BioSpec, Bartlesville, OK, USA), for 4 min at full speed. An equal volume of isopropanol and 0.1 volume of 3 M sodium acetate (pH 5.5) were added and gently mixed, and the mixture was stored at −20°C overnight (59). The cell pellets were precipitated with ethanol, followed by DNase treatment using the Baseline-ZERO DNase kit (Epicentre Technologies, Madison, WI, USA), and RNA was purified using the MEGAClear kit (Thermo Fisher Scientific), based on the manufacturer’s instructions. RNA yield and quality were assessed using the Bioanalyzer 2100 with the RNA 6000 Nano assay reagent kit from Agilent (Santa Clara, CA), and samples were stored at −85°C until RNA sequencing (RNA-seq), using Illumina HiSeq 2000 technology, at the Beijing Genomics Institute.

### Quantitative PCR assays.

Bacterial growth on xylan and pectin cannot be measured by changes in OD_600_, due to the cloudy nature of the medium containing these substrates. Therefore, qPCR assays were developed so that each strain could be enumerated separately, or collectively when cocultured, during growth on these insoluble substrates. To determine the target genes for the qPCR assays, a comparison of the complete B. hungatei MB2003 and B. proteoclasticus B316^T^ genomes to identify unique and conserved genome regions was conducted using differential BLAST analysis (60). The specificity of each primer/probe assay was demonstrated by qPCR, using the primers listed in Table 4. qPCR (total reaction volume, 20 μl) was performed using KAPA SYBR Fast qPCR kit master mix (2×) universal (KAPA Biosystems, Wilmington, MA, USA), 0.1 μM TaqMan sense primer, 0.1 μM TaqMan antisense primer, 0.1 μM TaqMan probe, and ≤20 ng of template DNA. qPCR amplifications were performed on a Rotor-Gene 6000 real-time rotary analyzer (Corbett Life Science, Qiagen), using the following qPCR program: enzyme activation at 95°C for 10 min, initial denaturation at 95°C for 10 s, and then 40 cycles of 95°C for 30 s, annealing at 56°C for 30 s, and extension at 72°C for 1 min per kilobase of DNA template. The absence of nonspecific signals was evaluated by melt curves for each qPCR between 72°C and 95°C. The melt curve temperature protocol was 1°C per step, with a wait of 90 s for premelt conditioning on the first step and 5 s for each step thereafter. The Rotor-Gene 6000 series software (Corbett Life Science) was used to generate the melt curves and to determine threshold cycle (*C_T_*) values. Confidence intervals were calculated by Student's *t* test, where the statistical significance was set at a *P* value of <0.05. All *C_T_* values are averages of at least three repetitions. The specificity of each qPCR amplicon was further confirmed by gel electrophoresis, showing a single band of the correct size.

**TABLE 4:** TaqMan primers/probes used to enumerate culture growth

Primer/probe	Specificity	Gene annotation	Locus tag	Amplicon size (bp)	Sequence
buk_Forward	B316^T^ and MB2003	Butyrate kinase (*buk*)	bpr_I2323	90	5′-ACCTTGGAACCAACGATATGAG-3′
buk_Reverse					5′-GGTAAATGAAAGCGTCACGAAC-3′
buk_Probe					5′-TGTGTGATGAGGGCAACGAGAAGG-3′
CBD_Forward	B316^T^	Cell-wall-binding-domain-containing protein	bpr_I0264	86	5′-CGCTACAGAGGGAACTGAAATAG-3′
CBD_Reverse					5′-CCTCAAGGCTCTCATCAACTAC-3′
CBD_Probe					5′-CTGTGGAAGCATCAAGCGCAGC-3′
Arf51C_Forward	MB2003	α-l-Arabinofuranosidase (*arf51C*)	bhn_I1532	88	5′-GCAAAGGTATGTTTGGACTGTT-3′
Arf51C_Reverse					5′-AAACTACGGTTCTCAAGCATTTC-3′
Arf51C_Probe					5′-TGCATGAAGACCACCGTCAAGACC-3′

### Fermentation end product analysis.

The VFA production profiles of *Butyrivibrio* strains were determined by quantification of acetic, butyric, and propionic acids and the branched chain VFAs isobutyric acid and isovaleric acid, which were used as indicators of growth and insoluble polymer substrate utilization of the *Butyrivibrio* strains (61). The *Butyrivibrio* cells for use as inocula were cultured as described previously and passaged three times. Cultures were then inoculated, in triplicate (0.5% [wt/vol] inoculum), into Hungate tubes containing 9.5 ml of RM02 medium and 0.5% (wt/vol) insoluble substrate and were grown anaerobically for 48 h at 39°C, with gentle horizontal shaking. The insoluble substrates analyzed were pectin, inulin, cellulose, dextrin, starch, and xylan. Uninoculated media supplemented with 0.5% cellobiose (wt/vol) and cultures grown with cellobiose (0.5% [wt/vol]) as the sole substrate were used as controls. Samples were analyzed using a Hewlett Packard/Agilent 6890 GC gas chromatograph system (GMI, Ramsey, MN, USA) with flame ionization detection. A HP-PLOT molecular sieve capillary column (30 mm by 0.53 mm [inner diameter] by 25 μm [film]; Agilent Technologies) was used for separation, with a column helium flow rate of 5.5 ml/min.

An additional method (62) was used to derivatize formic, lactic, and succinic acids, which could not be detected using the VFA method described above and are important fermentation products used to characterize *Butyrivibrio* strains phenotypically. The internal standard used was 2-ethylbutyric acid (Sigma-Aldrich), and samples were derivatized with *N*-methyl-*N-t*-butyldimethylsilyltrifluoroacetamide (MTBSTFA) (Sigma-Aldrich). Samples were analyzed using a Shimadzu GC-2010 Plus gas chromatograph with a helium ionization detector, and a MXT-Msieve5A PLOT capillary column (30 mm by 0.53 mm [inner diameter] by 50 μm [film]; Restek Corp., Bellefonte, PA, USA) was used for separation.

### High-pressure ion chromatography.

The monosaccharides released during *Butyrivibrio* monoculture and coculture growth were determined using a HPIC method (55). Culture samples from the eight time intervals (0, 2, 4, 6, 8, 10, 12 and 16 h) were used for analysis, and the HPIC system used was the DIONEX ICS-5000 system (Thermo Fisher Scientific). Data analysis was performed with Chromeleon software (Thermo Fisher Scientific). Using a two-tailed, unpaired Student's *t* test, differences between sample means were considered statistically significant if the *P* value was <0.05. Quantitative analyses were carried out using standard solutions of the monosaccharides arabinose (Sigma-Aldrich), fucose (Sigma-Aldrich), galactose (Sigma-Aldrich), glucose (VWR International Ltd., Poole, UK), rhamnose (VWR), and xylose (VWR).

### Growth curves for *Butyrivibrio* strains grown on mixtures of the component sugars of xylan and pectin.

B. hungatei MB2003 and B. proteoclasticus B316^T^ were cultured anaerobically, under a CO_2_ atmosphere, at 39°C in prewarmed RM02 medium supplemented with either 0.5% (wt/vol) xylan from oat spelt or apple pectin component sugars as the main carbohydrate source. The monosaccharide components (mole percent) of xylan were d-glucose (15%), l-arabinose (10%), and d-xylose (75%), as described (63). The monosaccharide components (mole percent) of pectin were d-galacturonic acid (70%), d-glucose (5%), d-galactose (8%), l-arabinose (2%), l-fucose (9%), and l-rhamnose (3%), as described (64). All monosaccharides were obtained from Sigma-Aldrich. Culture purity was verified via wet mounting and Gram staining. Cells were subcultured twice consecutively in triplicate with the respective growth medium, to ensure complete adaptation to the carbohydrate growth source. The OD_600_ was monitored using an Ultrospec 1100 Pro spectrophotometer (GE Healthcare) over a period of 24 h.

### Differential gene expression.

Total RNA libraries of 200-bp inserts were sequenced with Illumina HiSeq 2000 technology at the Beijing Genomics Institute. Paired-end sequencing with 90-bp reads was performed. Sequence data from the Beijing Genomics Institute had been filtered using DynamicTrim (65) to remove reads containing ≥10% unreadable bases or ≥20% low-quality (≥Q20) bases, as well as any reads with quality scores of ≤Q28, adapter contamination, or duplicate read pairs, and the quality of the sequence data before analysis was assessed using FastQC (66). Bowtie 2 (67) was used with default parameters to remove any sequence reads aligning with rRNA, tRNA, and noncoding RNA sequences. Reference-based transcriptome assembly (68, 69) was performed because high-quality genome sequences were available for both B. hungatei MB2003 (20) and B. proteoclasticus B316^T^ (14). Rockhopper (70, 71) was used with the remaining reads to identify differential expression in monoculture and coculture growth of B. hungatei MB2003 and B. proteoclasticus B316^T^ on xylan and pectin separately. The cutoffs used for a gene to be considered statistically a DEG in each bacterium in the monoculture versus coculture environments were a *Q* value of ≤0.05 and a log_2_ ≥2-fold change in expression. An overview of the RNA-seq *in silico* analysis is presented in Fig. S2.

### Statistical analysis of RNA-seq data.

Standard univariate and multivariate statistical tests were performed using R software to analyze the RNA-seq data sets (Table 1; also see Fig. S3 and Data Set S1). The Rockhopper output, containing the total number of aligned reads, also calculated the reads per kilobase of gene per million reads mapped (RPKM). A null hypothesis test based on a negative binomial distribution model was then performed (72, 73). Multiple tests were used to determine differential gene expression; *Q* values (or adjusted *P* values) that control for the FDR using the Benjamini-Hochberg procedure were also reported (74). Rockhopper was run for each set of FASTQ files separately, and the output data were grouped and compared, based on the treatments (xylan and pectin) relative to the growth conditions (monoculture versus coculture), for B. hungatei MB2003 and B. proteoclasticus B316^T^ separately.

Two forms of multidimensional scaling (75) were used to analyze the RNA-seq data, namely, nonmetric multidimensional scaling (76, 77) and metric scaling in the form of principal-coordinate analysis (PCoA) (78, 79). Bray-Curtis distances of the normalized counts were used to measure pairwise dissimilarity (80–82). Permutation multivariate analysis of variance (ANOVA) used distance matrices to define sources of variation among samples (83, 84). Correspondence analysis (CA) was utilized to explore the associations between groupings of samples (85). To test the strength of the *P* and *Q* values generated by the Rockhopper software, the Kruskal-Wallis ANOVA by ranks of the *P* values was performed on the normalized counts independently, to calculate the adjusted *P* values (86). This analysis incorporated the ANOVA *t* test, which is a nonparametric test investigating whether samples originated from the same distribution (87).

The full data set consisted of the merged monoculture and coculture transcripts of B316^T^ and MB2003 for both xylan and pectin growth conditions. Resulting *P* values from the ANOVA and *t* tests were corrected for multiple comparisons using the Benjamini-Hochberg method (74), into *Q* values. These *P* values from all statistical analyses were merged together and compared with *P* and *Q* values from the Rockhopper software for the xylan and pectin subsets separately, to determine significantly differentially expressed genes that complied with all analyses (Benjamini-Hochberg-method-adjusted ANOVA *t* test, *P* < 0.05; Benjamini-Hochberg-method-adjusted Kruskal-Wallis ANOVA by ranks, *P* < 0.05). Conservative thresholds of FDR *Q* value of <0.05, Kruskal-Wallis-test-adjusted *P* value of <0.05, and ≥2-fold log_2_-transformed signal intensity difference were used to define DEGs (Fig. S3). The numbers of significant DEGs (log_2_ ≥2-fold difference) for the monoculture and coculture conditions obtained using the FDR (*Q* < 0.05) and Kruskal-Wallis test (*P* < 0.05) cutoffs are shown in Table 1.

### Accession number(s).

Annotated B. hungatei MB2003 and B. proteoclasticus B316^T^ genomes were submitted to GenBank under GenBank accession numbers CP017831, CP017830, CP017832, CP017833, CP001810, CP001811, CP001812, and CP001813. The RNA-seq data have been deposited in NCBI Gene Expression Omnibus (GEO) and are accessible through GEO accession number GSE120544.

## Supplementary Material

Supplemental file 1

Supplemental file 2

Supplemental file 3

Supplemental file 4
