# Supplementary material for: Butyrivibrio hungatei MB2003 Competes Effectively for Soluble Sugars Released by Butyrivibrio proteoclasticus B316T during Growth on Xylan or Pectin
Source: Appl Environ Microbiol. 2019 Jan 23;85(3):e02056-18. doi: 10.1128/AEM.02056-18 (PMC6344614; doi:10.1128/AEM.02056-18)
Supplement: Supplemental file 1 [file acf34b743e82a277af290b2104247971_AEM.02056-18-s0001.pdf]

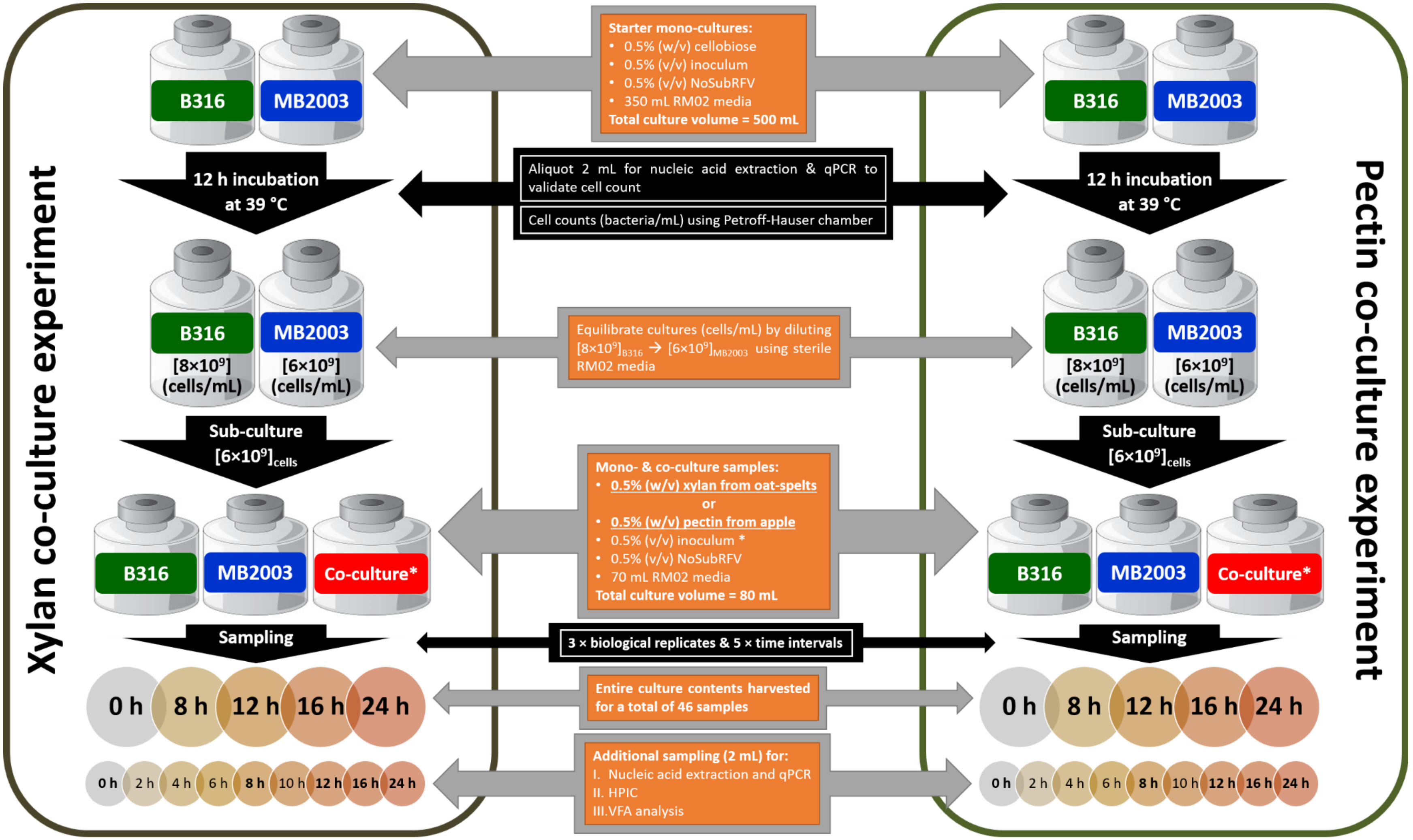

Fig. S1. Overview of *B. hungatei* MB2003 and *B. proteoclasticus* B316^T co-culture growth experiment. * 0.5% (v/v) co-culture inoculum comprised of 0.25% (v/v) *B. hungatei* MB2003 and 0.25% (v/v) *B. proteoclasticus* B316^T inocula.

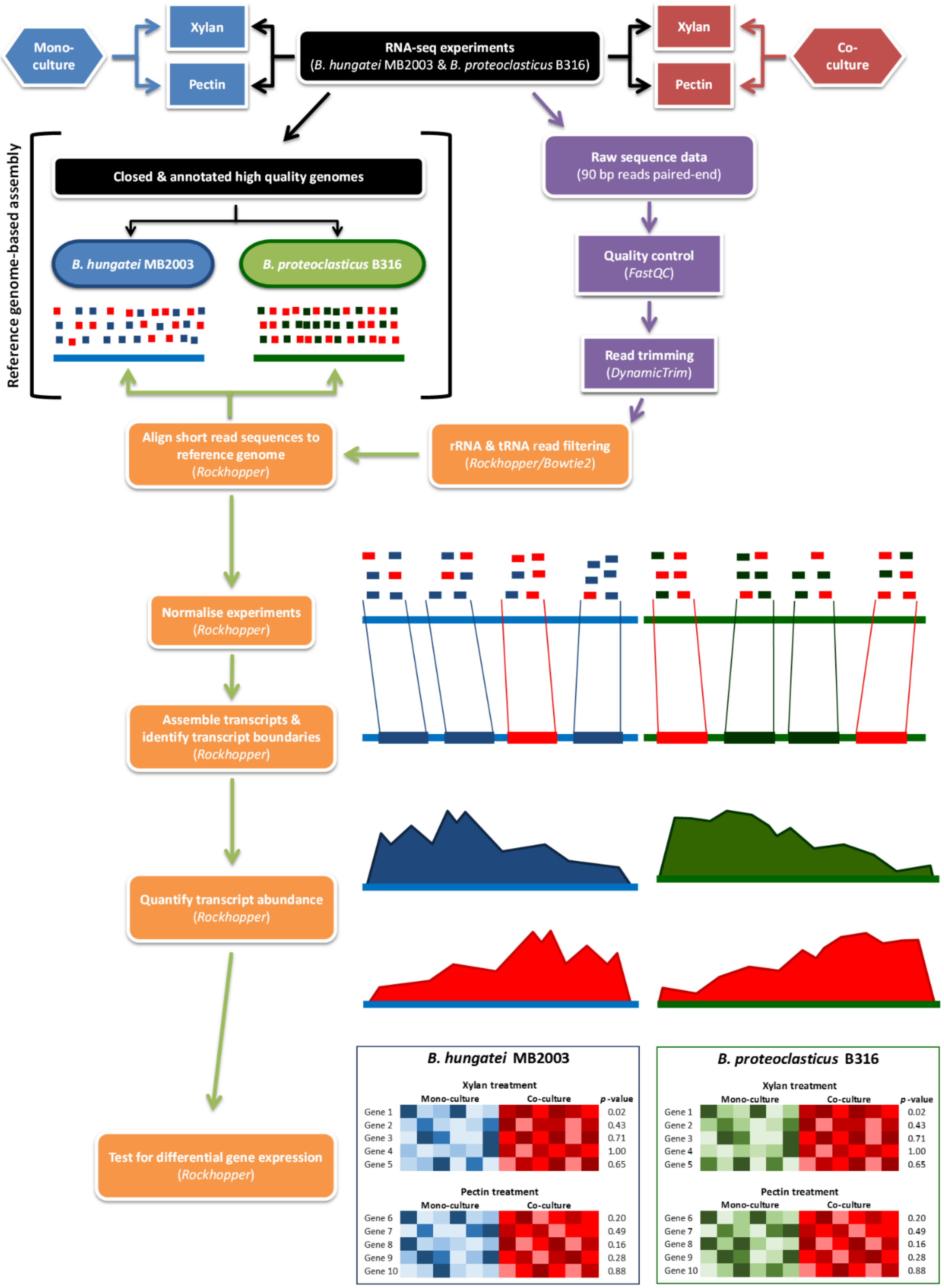

Fig. S2. Overview of the workflow for RNA-seq *in silico* analysis. Software and programmes used at each stage are *italicized* and shown in brackets.

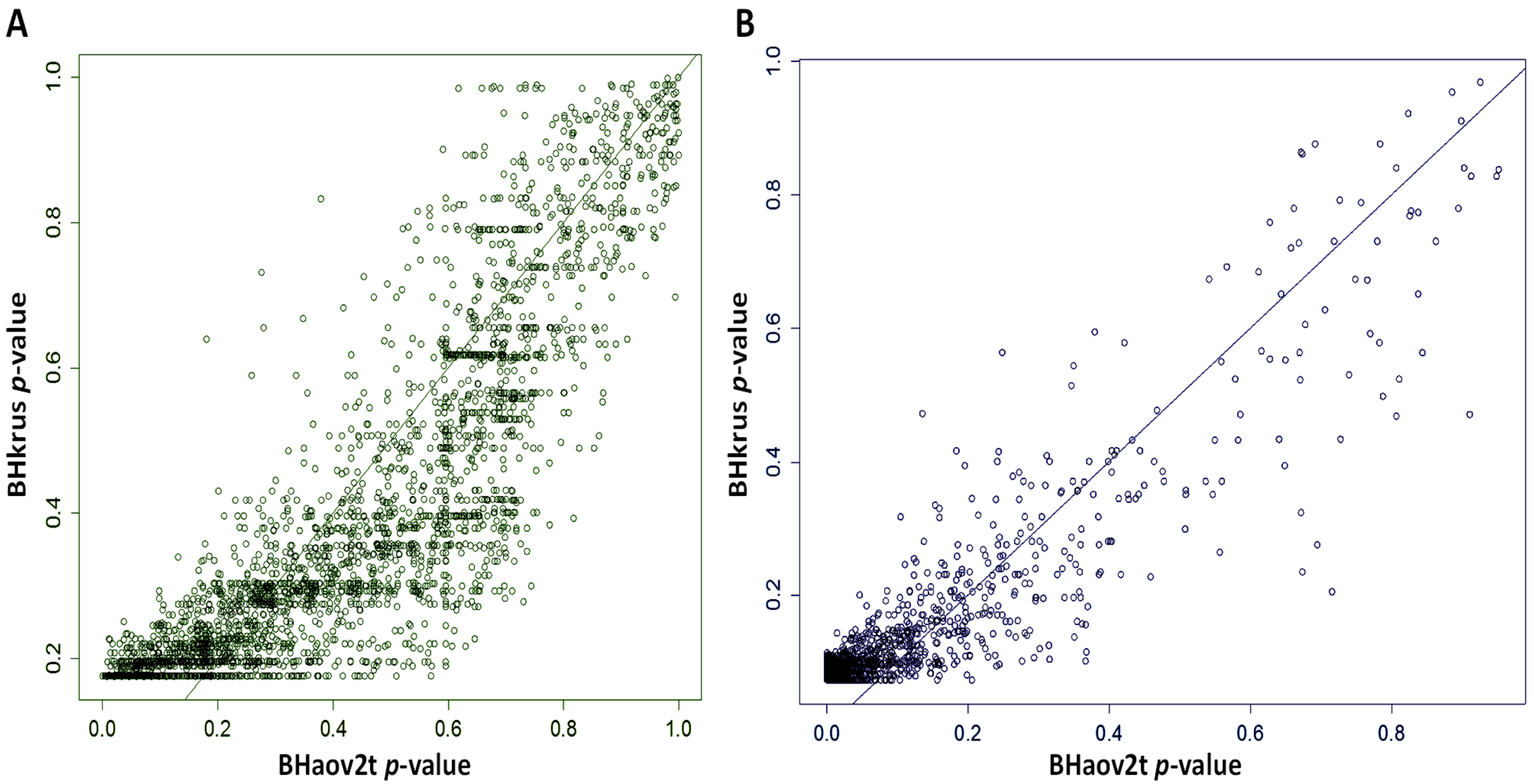

Fig. S3. Ordinate plots comparing BH-adjusted ANOVA and BH-adjusted KW analyses. A, *B. proteoclasticus* B316^{T} and B, *B. hungatei* MB2003 xylan and pectin complete transcriptome datasets. Abbreviations: BHaov2t *p*-value, Benjamin Hochberg adjusted ANOVA *t*-test *p*-values; BHkrus *p*-value, Benjamin Hochberg adjusted KW analysis of variance by ranks *p*-values.

Table S1. VFA analysis of mono- and co-cultures of *B. hungatei* MB2003 and *B. proteoclasticus* B316^T grown on xylan and pectin.

Substrate	Culture	VFA (mM) *					
		Acetate	SD	Butyrate	SD	Formate	SD
Xylan	*B. hungatei* MB2003	0.72	0.22	0.12	0.00	0.23	0.26
	B. proteoclasticus B316^T	1.38	0.36	1.94	0.04	6.14	0.04
	Co-culture	2.03	0.00	2.07	0.00	6.63	0.21
Pectin	*B. hungatei* MB2003	0.65	0.68	0.36	0.07	0.98	0.04
	B. proteoclasticus B316^T	6.73	0.00	0.44	0.00	6.25	0.04
	Co-culture	4.85	0.29	0.60	0.07	4.85	0.63

*, The numbers represent the change in acetate, butyrate and formate production between inoculation and 12 h post inoculation, and are the means of three replicate cultures. SD, standard deviation of the mean.
